# Polymorphisms in genes of interleukin 12 and its receptors and their association with protection against severe malarial anaemia in children in western Kenya

**DOI:** 10.1186/1475-2875-9-87

**Published:** 2010-03-29

**Authors:** Lyna Zhang, Donald Prather, Jodi Vanden Eng, Sara Crawford, Simon Kariuki, Feiko ter Kuile, Dianne Terlouw, Bernard Nahlen, Altaf A Lal, Laurence Slutsker, Venkatachalam Udhayakumar, Ya Ping Shi

**Affiliations:** 1 Malaria Branch, Division of Parasitic Diseases, National Center for Zoonotic, Vector-Borne & Enteric Diseases, Coordinating Center for Infectious Diseases, Centers for Disease Control and Prevention, Atlanta, GA 30341, USA; 2 Office of Public Genomics, Coordinating Center for Health Promotion, Centers for Disease Control and Prevention, Atlanta, GA 30333, USA; 3 Kenya Medical Research Institute, Kisumu, Kenya; 4 Liverpool School of Tropical Medicine, Liverpool L3 5QA, UK

## Abstract

**Background:**

Malarial anaemia is characterized by destruction of malaria infected red blood cells and suppression of erythropoiesis. Interleukin 12 (IL12) significantly boosts erythropoietic responses in murine models of malarial anaemia and decreased IL12 levels are associated with severe malarial anaemia (SMA) in children. Based on the biological relevance of IL12 in malaria anaemia, the relationship between genetic polymorphisms of *IL12 *and its receptors and SMA was examined.

**Methods:**

Fifty-five tagging single nucleotide polymorphisms covering genes encoding two *IL12 *subunits, *IL12A *and *IL12B*, and its receptors, *IL12RB1 *and *IL12RB2*, were examined in a cohort of 913 children residing in Asembo Bay region of western Kenya.

**Results:**

An increasing copy number of minor variant (C) in *IL12A *(rs2243140) was significantly associated with a decreased risk of SMA (*P *= 0.006; risk ratio, 0.52 for carrying one copy of allele C and 0.28 for two copies). Individuals possessing two copies of a rare variant (C) in *IL12RB1 *(rs429774) also appeared to be strongly protective against SMA (*P *= 0.00005; risk ratio, 0.18). In addition, children homozygous for another rare allele (T) in *IL12A *(rs22431348) were associated with reduced risk of severe anaemia (SA) (*P *= 0.004; risk ratio, 0.69) and of severe anaemia with any parasitaemia (SAP) (*P *= 0.004; risk ratio, 0.66). In contrast, AG genotype for another variant in *IL12RB1 *(rs383483) was associated with susceptibility to high-density parasitaemia (HDP) (*P *= 0.003; risk ratio, 1.21).

**Conclusions:**

This study has shown strong associations between polymorphisms in the genes of *IL12A *and *IL12RB1 *and protection from SMA in Kenyan children, suggesting that human genetic variants of IL12 related genes may significantly contribute to the development of anaemia in malaria patients.

## Background

Malaria continues to be a major global public health burden, causing 250 million clinical cases and nearly one million deaths annually, of which 85% are children under the age of five. About 86% of malaria cases and over 91% of malaria deaths worldwide occur in sub-Saharan Africa [1,2]. Cerebral malaria and severe malarial anaemia (SMA) are two major syndromes causing malaria-related mortality in children. The pattern of these two severe forms varies depending on the intensity of transmission: cerebral malaria is more common in older children in areas with lower intensity of transmission, whereas SMA is often seen in children below two years of age in areas with intense transmission [3], such as western Kenya, where *Plasmodium falciparum *infection is most common [4].

The human host response to *P. falciparum *can vary. While some infected individuals die of severe malaria, others survive, and still others are infected without becoming severely ill. It has been estimated that host genetic factors account for ~25% of the risk of severe malaria [5]. Malaria has exerted significant pressure on the human genome to select for mutations conferring refractoriness to malaria [6]. This is illustrated by several well studied mutations that protect against different forms of malaria, including sickle cell allele, Duffy mutations, and alpha thalassaemia [7]. An increasing amount of published information has documented a number of genetic associations with malaria susceptibility. The majority of these concern genes coding for molecules involved in physiology of red blood cells or in the host immune response[8]. It is only beginning to understand the human genetic factors that determine resistance to malaria.

Malarial anaemia is characterized by destruction of malaria infected red blood cells and suppression of erythropoiesis [9-11]. Although the mechanisms of erythropoietic suppression associated with pathogenesis of malarial anaemia are poorly understood, studies in murine models of malarial anaemia have demonstrated that interleukin 12 (IL12) significantly boosts erythropoietic responses by enhancement of erythroid progenitor expansion and immune regulation [12-15], and administration of IL12 corrects malarial anaemia [12,16,17]. Moreover, several immuno-epidemiological studies conducted in malaria endemic areas have shown that decreased IL12 levels are associated with an increased risk of SMA in children [18-20]. Taken together, these findings suggest that reductions of IL12 in malaria infection could lead to SMA by suppression of erythropoiesis.

IL12 is a pro-inflammatory cytokine with a pleiotropic effect as a potent immunoregulatory molecule and haematopoietic growth factor in infections with *Plasmodium *parasites [14]. It is a disulfide-linked heterodimer composed of a 35 kD subunit encoded by *IL12A *and 40 kD subunit encoded by *IL12B *and exerts its biological effects through binding to heterodimeric receptors encoded by *IL12RB1 *and *IL12RB2*. The region containing *IL12B*, chromosome 5q31-q33, is linked to *P. falciparum *parasite density in linkage analysis studies [21,22]. A functional promoter variant in *IL12B*, IL12Bpro (rs17860508) is associated with protection against cerebral malaria in children [23]. These results suggest that IL12 is an important candidate gene in host resistance to severe malaria.

Given the biological relevance of IL12 in malarial anaemia and the genetic linkage of *IL12B *in malaria infection and severe disease (cerebral malaria), the potential associations of differences in genes encoding two human IL12 subunits, *IL12A *and *IL12B*, and receptors, *IL12RB1 *and *IL12RB2*, in the risk of developing SMA has been investigated by retrospectively genotyping samples collected from an infant cohort study, Asembo Bay Cohort Project (ABCP) [24]. This study presents findings of the first significant genetic association within the *IL12A *and *IL12RB1*, which are associated with protection against SMA among young children in western Kenya.

## Methods

### Participants

This study was part of Asembo Bay Cohort Project, an immuno-epidemiologic birth cohort project conducted from June 1992 through September 1996. Characteristics of this study have been detailed elsewhere [24]. Briefly, the study was carried out in a rural area of western Kenya where more than 95% of the residents belong to the Luo ethnic group, malaria transmission is holoendemic, and severe anaemia is a major cause of malaria-related mortality in children less than two years of age. Pregnant women were enrolled in their second or last trimester; their newborn babies were enrolled soon after birth and followed up to three to five years of life. Village monitors visited each mother-child pair every two weeks for clinical observation. At monthly intervals (and between intervals when children were sick), blood samples were obtained for measurement of parasitaemia and haemoglobin concentrations. Resulting data, along with time to first documented malaria infection, parasite density of first infection, and cumulative treatment with sulphadoxine/pyrimethamine were available as part of routine data collection. For the current genetic association study, longitudinal clinical information up to two-year follow-up from the time of enrollment was used since this age group accounted for most of the observed malarial anaemia [25].

Inclusion criteria for children in this genetic association study included having at least 1 monthly follow-up visit up to two years of age and availability of sample. Mean numbers of visits including both monthly follow-ups and sick visits during first two years of life were 19.8 (SD = 11.5) for the included children. Additionally, for genetic association analyses, it is important that all participants are genetically unrelated individuals. Therefore, only children from singleton births or one of the twins were included in the study. If more than one family member (siblings) was eligible based on the above criteria, only the first child in birth order was included in the study.

Informed written consent was obtained from parents/guardians of all study participants. This study was approved by the Institutional Review Board of the Kenya Medical Research Institute and Centers for Disease Control and Prevention.

### Phenotypic definitions

A classification scheme [26], based on previous epidemiological analysis of the same cohort [25], was used in this study. SMA was defined as a haemoglobin concentration of less than 6 g/dL and the presence of *P. falciparum *greater than 10,000/μl. Three other subphenotypes used in this study were: severe anaemia (SA) (Hb < 6 g/dL), high-density parasitaemia (HDP) (> 10,000 *P. falciparum *parasites/μl), and severe anaemia with any level of parasitaemia (SAP) (Hb < 6 g/dL with any detectable *P. falciparum *parasitaemia). All data points collected monthly and at sick visits for two years in children were used and the clinical phenotypes are presented as episode crude incidence/1,000 person-months.

### Single nucleotide polymorphism (SNP) selection

For tagging SNP (tagSNP) selection, LDSelect software at default settings (*r*^2 ^> 0.65) was used to select tagSNPs from all common variation within four IL12 related genes from a public database of SeattleSNPs [27]. LDSelect uses an efficient selection algorithm to select tagging SNPs based on linkage disequilibrium (LD) statistic r^2 ^and does not require direct haplotype inference [28]. At each round of selection, the binning algorithm identifies single SNP, which exceeds threshold r^2 ^with the maximum number of other SNPs, and sets this group of SNPs as a bin. Then each SNP within the bin is analysed to determine whether it exceeds the threshold r^2 ^with all other SNPs in the bin. All SNPs in a bin that meet this criterion are designated as tagSNPs. Only one tagSNP needs to be typed per bin. Binning criteria for tagSNP selection used in this study were minor allele frequency (MAF) cut-off of 10% and an r^2 ^threshold of 0.65. LD varies across different populations [29,30]; therefore, only genotype data for the African-American samples present in the database was used, as this population is most similar to the samples in this study from western Kenya. Fifty five tagSNPs, 7 in *IL12 A*, 10 in *IL12B*, 22 in *IL12RB1*, and 16 in *IL12RB2 *were selected. Many more SNPs were needed for *IL12RB1 *gene than for other genes tested due to its larger size and the genetic diversity.

### Genotyping

DNA was extracted from frozen blood pellets using a QiaAMP DNA purification kit (Qiagen). For each sample, 10 ng DNA was spotted into 384-well plates (ABgene), dried and frozen until just before use in genotyping assays. SNPs were genotyped using the MassARRAY^® ^iPLEX assay (Sequenom)[31]. Multiplex PCR primers (up to 29-plex) were designed in a region of approximately 100 base pairs around the SNP of interest and an extension primer was designed immediately adjacent to the SNP using MassArray Assay Design software (version 3.1). The single base primer extension step was performed using a standardized cycling programme. Primer extension products were dispensed on 384-well SpectroChip^®^s that were automatically read by a Compact™ MALDI-TOF mass spectrometer (Sequenom). Data analysis was performed on MassArray Typer software (version 3.4).

### Data quality control and statistical analysis

Genotypes were individually examined via a detailed quality control process involving duplicate calling of genotypes, control samples, evaluation of missing calls, and Hardy--Weinberg Equilibrium (HWE) testing. The iPLEX genotype clusters were manually checked. Only SNPs with > 90% calling rate and MAF > 2% were including in the final analysis. Each SNP was analysed using univariate methods and then multivariate Poisson regression analysis to ascertain the association between genotypes and malaria-associated morbidity. Generalized estimating equations and an independent working correlation structure to adjust for correlation between multiple visits from the same individual were used. In the multivariate Poisson regression, SNPs were independently evaluated as covariates using one of four genetic models: dominant ((AA + Aa) vs aa), recessive (AA vs (Aa + aa)), additive (aa vs Aa vs AA, which treat the marker as a continuous variable), and heterozygous advantage (Aa vs (AA + aa)) with three levels based on the most common allele frequency. Here, "A" refers the common allele. The results of the Poisson regression were reported as rate ratios (or risk ratios, RR). Multivariate models were adjusted for confounders, sickle cell type and treatment with anti-malarial drugs since these two factors were significantly associated with all four clinical phenotypes in univariate analysis (Table 1). Data was analysed using SAS software package (version 9.1). Bonferroni adjusted critical alpha level of 0.007 for *IL12A*, 0.006 for *IL12B*, 0.005 for *IL12RB1*, and 0.004 for *IL12RB2 *were used. Haploview was used to obtain values for LD and perform haplotype block analysis of SNPs in the same gene [32,33]. D prime values (D') were calculated as statistical values for pairwise LD analysis between SNPs.

**Table 1:** Characteristics of 913 infants selected from Asembo Bay Cohort Project

	Subjects, no. of any incidence (%)^a^								
	Total	SA^b^	*P*	HDP^c^	*P*	SAP^d^	*P*	SMA^e^	*P*^f^
Sex									
Male	441	213 (48)	.03	357 (81)	.33	192 (44)	.03	77 (18)	.33
Female	472	194 (41)		370 (78)		172 (36)		71 (15)	
Sickle cell type									
AS	147	51 (35)	.01	112 (77)	.02	46 (31)	.03	13 (9)	.008
AA	656	316 (48)		547 (83)		280 (43)		122 (17)	
SS	25	13 (52)		17 (68)		12 (48)		2 (8)	
Anti-malarial use									
Yes	786	371 (47)	< .0001	661 (84)	< .0001	331 (42)	.0006	137 (17)	.01
No	127	36 (28)		66 (52)		33 (26)		11 (9)	
Placental blood parasitaemia									
Yes	210	84 (40)	.05	161 (77)	.11	76 (36)	.09	35 (17)	.93
No	481	232 (48)		394 (82)		207 (43)		79 (16)	
Maternal peripheral parasitaemia									
Yes	296	121 (41)	0.1	230 (78)	.25	109 (37)	.17	57 (19)	.11
No	598	279 (47)		484 (81)		249 (42)		90 (15)	
Birth weight < 2500 g or gestation age < 37 weeks									
Yes	82	35 (43)	.58	59 (72)	.03	31 (38)	.54	15 (18)	.58
No	636	292 (46)		522 (82)		263 (41)		101 (16)	
Low birth weight (< 2500 g)									
Yes	71	29 (41)	.41	51 (72)	.05	25 (35)	.31	13 (18)	.61
No	651	299 (46)		532 (82)		270 (41)		104 (89)	
Pre-term birth (< 37 weeks gestation)									
Yes	29	11 (38)	.44	18 (62)	.01	10 (35)	.51	3 (10)	.37
No	846	382 (45)		685 (81)		343 (41)		141 (17)	

NOTE

^a ^Any incidence: at least once; displayed are frequency counts (percentage).

^b ^SA: Severe anaemia (Hb < 6 g/dL).

^c ^HDP: High density parasitaemia (> 10,000 *P. falciparum *parasites/μl).

^d ^SAP: Severe anaemia with any density parasitaemia (Hb < 6 g/dL with any detectable *P. falciparum *parasitaemia).

^e ^SMA: Severe malaria anaemia Hb < 6 g/d Land > 10,000 *P. falciparum *parasites/μl)

^f ^Chi-square p-values.

## Results

### Characteristics of study participants

Among 1,459 children enrolled in ABCP, 913 meeting inclusion criteria (62.6%) were selected for genotyping. In this study, incidence of at least one SMA episode in the same individual was 16.5% during the first two years of life, 44.5% for SA, 79.5% for HDP, and 40.0% for SAP. Only sickle cell type and history of anti-malarial use were significantly associated with all four clinical phenotypes of interest in the unadjusted analysis (Table 1). These two covariates were therefore included in the final model of multivariate Poisson regression analysis. Other covariates, such as bed net use, amount of rain in previous 120 days, parasitaemia in the mothers at the time of delivery, and low birth weight (< 2500 g) or pre-term birth (< 37 weeks gestation), were not significantly associated with all four clinical phenotypes although parasitaemia in mother was associated with SA only and low birth weight and pre-term birth were associated with HDP.

### Genotyping

Fifty-five tagSNPs were selected for genotyping from genes encoding *IL12 A*, *IL12 B*, *IL12RB1*, and *IL12RB2*. Eight SNPs were not polymorphic in this population, and nine did not perform sufficiently in the multiplexed assay or pass quality control process (Table 2). Thirty-eight SNPs were included in the final analysis (Additional file 1).

**Table 2:** List of SNPs of *IL12A*, *IL12B*, *IL12RB1 *and *IL12BR2 *tested in this study

SNP rs#	Gene(s)	Alleles	SNP position	MAF*	Role	AA change
rs2243113	*IL12A*	A/G	chr3:161188557	0.08	Promoter	-
rs582054	*IL12A*	T/A	chr3:161192695	0.21	Intron	-
rs583911	*IL12A*	A/G	chr3:161193084	0.11	Intron	-
rs2243128	*IL12A*	G/A	chr3:161193205	0.17	Intron	-
rs2243138	*IL12A*	A/T	chr3:161197044	0.47	Downstream	-
rs2243140	*IL12A*	T/C	chr3:161197241	0.11	Downstream	-
rs2243143	*IL12A*	A/G	chr3:161197496	0.45	Downstream	-
rs3212227	*IL12B*	A/C	chr5:158675537	0.39	3' UTR	-
rs3213119	*IL12B*	C/A	chr5:158676366	0.00	Coding exon	298 V/F
rs2195940	*IL12B*	C/T	chr5:158676930	0.25	Intron	-
rs3213103	*IL12B*	G/A	chr5:158679014	0.07	Intron	-
rs3213099	*IL12B*	C/G	chr5:158680114	0.02	Intron (boundary)	-
rs919766	*IL12B*	A/C	chr5:158680142	0.28	Intron (boundary)	-
rs2853694	*IL12B*	T/G	chr5:158681666	0.17	Intron	-
rs3213096	*IL12B*	C/T	chr5:158682907	0.00	Coding exon	33 V/I
rs2569253	*IL12B*	T/C	chr5:158683571	0.17	Intron	-
rs2569254	*IL12B*	C/T	chr5:158683827	0.06	Intron	-
rs3746190	*IL12RB1*	G/A	chr19:18031384	0.07	3' UTR	-
rs383483	*IL12RB1*	A/G	chr19:18032886	0.44	Intron (boundary)	-
rs438421	*IL12RB1*	C/T	chr19:18037086	0.30	Intron	-
rs2045386	*IL12RB1*	G/A	chr19:18043266	0.28	Intron	-
rs447171	*IL12RB1*	C/T	chr19:18045647	0.27	Intron	-
rs11575934	*IL12RB1*	T/C	chr19:18047618	0.14	Coding exon	214 Q/R
rs429774	*IL12RB1*	T/C	chr19:18047752	0.24	Intron (boundary)	-
rs17882031	*IL12RB1*	C/T	chr19:18048349	0.00	Intron	-
rs11575926	*IL12RB1*	C/T	chr19:18049408	0.00	Coding exon	156 R/H
rs11575925	*IL12RB1*	G/C	chr19:18053977	0.00	Coding exon	74 S/R
rs897751	*IL12RB1*	A/G	chr19:18054349	0.30	Intron	-
rs439409	*IL12RB1*	G/A	chr19:18054613	0.32	Intron	-
rs17884651	*IL12RB1*	C/A	chr19:18058626	0.00	Coding exon	3 P/Q
rs393548	*IL12RB1*	T/A	chr19:18058744	0.27	Promoter	-111
rs7544381	*IL12RB2*	C/T	chr1:67546881	0.49	Intron	-
rs17129749	*IL12RB2*	C/T	chr1:67552339	0.30	Intron	-
rs17129751	*IL12RB2*	G/A	chr1:67552503	0.04	Intron	-
rs2307147	*IL12RB2*	T/C	chr1:67559874	0.24	Coding exon	26 D/D
rs17129778	*IL12RB2*	A/T	chr1:67560279	0.12	Intron	-
rs17129792	*IL12RB2*	G/A	chr1:67565087	0.04	Coding exon	149 R/Q
rs1495963	*IL12RB2*	C/T	chr1:67567907	0.32	Coding exon	238 S/S
rs2307149	*IL12RB2*	A/T	chr1:67568038	0.06	Intron (boundary)	-
rs17838054	*IL12RB2*	G/A	chr1:67589869	0.00	Intron	-
rs2307145	*IL12RB2*	G/C	chr1:67606115	0.11	Coding exon	426 Q/H
rs2307153	*IL12RB2*	G/A	chr1:67606231	0.004	Coding exon	465 G/D
rs6685568	*IL12RB2*	A/G	chr1:67627885	0.45	Intron	-
rs2270614	*IL12RB2*	A/G	chr1:67628609	0.17	Intron	-
rs2229546	*IL12RB2*	C/A	chr1:67634108	0.46	Coding exon	779 P/P
rs17838066	*IL12RB2*	T/G	chr1:67634194	0.00	Coding exon	808 L/R

*MAF observed in this study.

### Association of gene polymorphisms in IL12 and its receptor with clinical phenotypes

This study identified an association with SMA for two SNPs, rs2243140 of *IL12A *and rs429774 of *IL12RB1*, both of which were associated with protection from SMA (Table 3). Specifically, for rs2243140 of *IL12A*, an increasing copy number of minor allele C (TT vs TC vs CC) was significantly associated with a reduced risk of SMA (*P *= 0.006). For rs429774 of *IL12RB1*, individuals possessing two copies of minor variant C appeared to be associated with a reduced risk of SMA (*P *= 5 × 10^-5^). The risk for developing SMA over time based on risk ratios calculated from multivariate Poisson regression was reduced by 82% (95% CI: 31%-95%) if carrying two copies of minor allele (CC) of rs429774 compared with those carrying one or no copies of the C allele. No statistically significant interaction between these two SNPs (*P *= 0.38) was found. These two minor alleles are common in the population studied here, 11% and 24%, for rs2243140 and rs429774, respectively (Table 2).

**Table 3:** Protective effect of polymorphisms in the genes of *IL12A *and its receptor, *IL12RB1*, against severe malaria anaemia

	Severe malaria anaemia episodes crude incidence/1000 person-months			Adjusted risk ratio (95% CI)^*a*^		
*IL12 A*-rs2243140^b^	TT	TC	CC	CC vs TT	TC vs TT	*P*
	12.2	7.7	0	0.28 (0.10-0.79)	0.52 (0.31-0.89)	.006
*IL12RB1*-rs429774^c^	TT	TC	CC	CC vs (TT+TC)		*P*
	12.2	11.2	2.8	0.18 (0.05-0.69)		.00005

NOTE.

^a ^Adjusted by sickle cell type and treatment with anti-malarial drugs.

^b ^An additive genetic model used for *IL12A*-rs2243140, which treats the marker as a continuous variable and is extrapolated based on Poisson model.

^c ^A dominant model used for the second marker, *IL12RB1*-rs429774.

Another SNP in *IL12A*, rs2243138, was associated with protection from SA (*P *= 0.004) and SAP (*P *= 0.004) (Table 4). The children homozygous for the rare allele (TT), of which the prevalence is 21.8% in this population, had significantly lower risk for developing SA (RR = 0.69, 95% CI: 0.53-0.89) and SAP (RR = 0.66, 95% CI: 0.49-0.89). A similar trend of association was seen with SMA, but was not statistically significant after adjusting for multiple comparisons (Additional file 1).

**Table 4:** Protective effect of polymorphisms in the genes of *IL12A *and its receptor, *IL12RB1*, against severe anaemia, severe anaemia with any parasitaemia and high-density parasitaemia

	Crude incidence/1000 person-months			Adjusted risk ratio (95% CI)^a^	
*IL12 A*-rs2243138^b^	AA	AT	TT	TT vs (AA + AT)	*P*
Severe anaemia episodes	38.3	41.3	30.3	0.69 (0.53-0.89)	.004
Severe anemia with any parasitaemia episodes	32.8	35.8	24.5	0.66 (0.49-0.89)	.004
*IL12RB1*-rs383483^c^	AA	AG	GG	AG vs (AA+GG)	*P*
High-density parasitaemia episodes	155.2	172.8	146.8	1.21 (1.07-1.36)	.003

NOTE.

^a ^Adjusted by sickle cell type and treatment with anti-malarial drugs.

^b ^A dominant model used for *IL12 A*-rs2243138.

^c ^A heterozygous advantage model used for *IL12RB1*-rs383483.

Another SNP in *IL12RB1*, rs383483, was associated with HDP (*P *= 0.003) (Table 4). Having an AG genotype, which is common (45.8%) in this population, was significantly associated with susceptibility to HDP at 1.21 (95% CI: 1.07-1.36) times greater risk compared with those with AA or TT genotypes. No association between SNPs from *IL12B *and *IL12RB2 *and malaria-related morbidity was identified.

**Figure 1:**
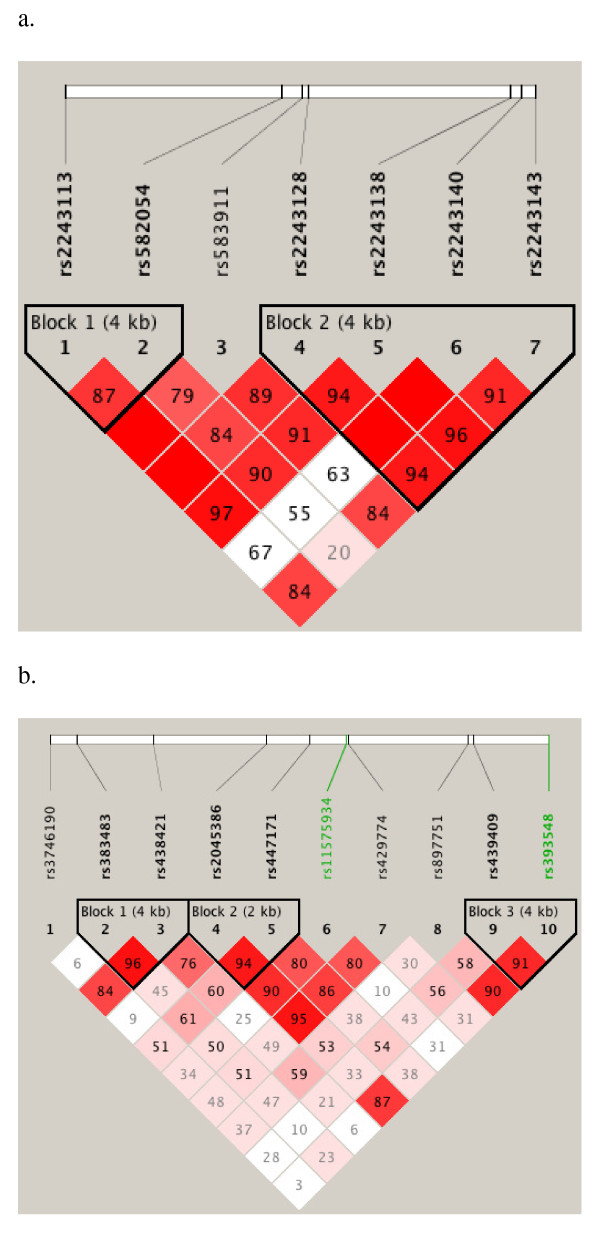
**Pairwise linkage disequilibrium (LD) plots of *IL12A *(a) and *IL12RB1 *(b) polymorphisms**. Data from all 913 participants are included. If LOD ≥ 2, red denotes D primer (D') = 1; shades of pink/red, 0 < D' < 1. If LOD < 2, white indicates 0 < D' < 1. The number in each box = 100 × D' value, except D' = 1 (the box is empty). The plots were generated using Haploview software.

Because the single SNP regression analysis demonstrated that multiple sites within *IL12A *and *IL12RB1 *genes are significantly associated with protection from severe malaria anaemia (Additional file 1), analysis of LD in these two genes (Figure 1) was performed respectively. The two hit SNPs of *IL12A*, rs2243140 and rs2243138, were in high LD (D' = 0.98), but SNPs of *IL12RB1*, rs383483 and rs429774, were not (D' = 0.47). This suggests that the associations shown by SNPs rs2243140 and rs2243138 of *IL12A *may be related whereas the rs383483 and rs429774 of *IL12RB1 *are independent.

## Discussion

In this paper, significant associations between polymorphisms within genes encoding the *IL12A *and *IL12RB1 *and protection against SMA are identified. The data shows that a SNP located within the 7^th ^intron boundary of the *IL12RB1 *gene (rs429774) is associated with protection against developing SMA. In particular, children possessing two minor alleles (C) had an 82% decreased risk of SMA compared to those with one or two copies of the major allele (T); this remained significant after correction for multiple testing. The decreased risk for developing SMA is also associated with increasing minor allele (C) copy number in a SNP located within the downstream of the *IL12A *gene (rs2243140). Another two SNPs in *IL12A *(rs22431348) and *IL12RB1 *(rs383483) were associated with reduced risk of SA and SAP and with increased risk HDP, respectively. There is no evidence to support any significant association between *IL12B *genetic variants and SMA or the 3 other clinical phenotypes tested in this study. This suggests that common genetic variations within *IL12B *did not influence the severity of malaria in this Kenyan population, despite previous reported associations of IL12Bpro (rs17860508) with cerebral malaria in Tanzanian and Malian, but not Kenyan children [23,34], and of several other diseases with variation in IL12Bpro (rs17860508) and IL12B 3' UTR (rs3212227) [35]. A lack of significance in IL12B in this study may be the result of lack of power or small sample size; however, a recent study conducted in western African population also showed that there was no association between these two known *cis*-regulatory element polymophisms in *IL12B *and malaria parasitaemia [36].

This study was conducted in a large and clinically well-characterized cohort of children using a tagSNP method based on the following considerations. First, although methods have been developed to predict the functional impact of variants such as nonsynonymous coding SNPs, which introduce amino acid changes in their corresponding proteins [35], these SNPs represent a small subset of all common variants. Second, several genome wide association studies have indicated that variations in non-coding regions rather than nonsynonymous coding SNPs are likely to be causative to disease susceptibility or resistance in most instances [37]. Regulation of the protein products, rather than differences in the structure or function of the protein may be most important for disease risk [38]. Third, many SNPs show correlated genotypes, or LD, suggesting that a subset of all SNPs (known as tagSNPs) can be genotyped for disease association studies [39]. Lastly, several tagSNP methods have been developed to select a subset of SNPs that efficiently describes existing patterns of variation in candidate gene regions for subsequent genotyping and association analysis, without making assumption about the potential functional impact of each SNP [40]. An efficient set of tagSNPs to cover each candidate gene region, rather than simply focusing on a few putatively interesting SNPs have been selected, such that genotype at untyped SNPs can be accurately inferred from genotype at a tagSNP [28].

Previous immunological studies have shown that IL12 exerts its biological function via binding to its receptors. This study has identified associations of polymorphisms not only in *IL12 *related gene, *IL12A*, but also in its receptor, *IL12RB1 *with SMA and HDP with different effects. Two SNPs in the *IL12A *(rs2243140 and rs2243138) and one SNP in the *IL12RB1 *(rs429774) were associated with protection against SMA or SA whereas one SNP in the *IL12RB1 *(rs383483) was associated with susceptibility to HDP. There are a number of studies that examined the effect of IL12 on erythropoiesis and malarial anaemia in mouse models, in which IL12 was found to be extremely effective in correcting malarial anaemia [12-14]. The dramatic effect of IL12 treatment is mediated by enhancement of erythroid progenitor expansion in bone marrow and by immune regulation of IFN-γ, TNF-α and NO [16]. In humans, a decrease in IL12 production is also clearly associated with SMA [18-20]. Although it remains unknown how the noncoding polymorphic SNPs in *IL12A *and *IL12RB1 *identified in this study control or regulate the expression of IL12 and its receptors, the results from this study support the assertion that non-coding regions rather than nonsynonymous coding SNPs potentially have effects on disease outcomes [39]. The results from this study also suggest that the effects of IL12A and IL12RB1 on malaria disease outcomes most likely result from the long evolutionary history of *P falciparum *parasite within the human population.

The IL23 pathway may offer further insights on the genetic results from this study since IL23 and IL12 share the IL12p40 subunit encoded by *IL12B *and IL23 also binds to the IL12 receptor subunit encoded by *IL12RB1*. A recent study has shown that children with malarial anaemia had increased circulating IL23 and IL10 and decreased IL12 relative to healthy controls in Kenyan children, suggesting that IL23 and IL12 are counter-regulators in malaria anaemia [20]. The findings from this study that polymorphisms (3 SNPs) in *IL12A *and *IL12RB1*, but not in *IL12B*, have protective effects on SMA and one SNP in *IL12RB1 *has an opposite effect on HDP are interesting. It is possible that the genetic effects on disease outcomes observed in this study may occur at the phenotypic level of IL12 and IL23 systems, which may be influenced by the interaction of genetic variants in *IL12A*, *IL12RB1 *and *IL12B*. This hypothesis requires testing. Currently, whether the polymorphisms in *IL12A *and *IL12RB1 *observed in this study influence the level and expression of IL12, IL12 receptor and IL23 *in vitro *in relation to severity of malaria anaemia have being examined in Kenyan children.

This study included a large sample size and used longitudinal data for association analysis. However, it will be important to replicate these findings in additional African populations [41]. These efforts are complicated by the patterns of disease seen in regions of differing malaria transmission, but these studies are needed to allow us to further understand the most important pathways in successfully controlling infection by the malaria parasite and to assess the effects of racial differences, transmission intensity, and other environmental factors on the interaction of *IL12 *related gene and severe malaria anaemia. In addition, studies are needed to examine whether the polymorphisms observed in these genes influence the level and expression of IL12, IL12 receptor and IL23 *in vitro *in other African populations in relation to malarial anaemia.

## Conclusions

This study has shown strong associations between polymorphisms in the genes of *IL12A *and *IL12RB1 *and protection from SMA in Kenyan children, suggesting that human genetic variants of IL12 related genes may significantly contribute to the development of anaemia in malaria patients.

## Competing interests

The authors declare that they have no competing interests.

## Authors' contributions

LZ was responsible for tagSNP selection, carried out genotyping work and genetic data analysis, and wrote manuscript. DP conducted genotyping and genetic data analysis. JVE and SC performed statistical analysis. SK, FTK and DT implemented Asembo Bay Cohort Project including collection of clinical and epidemiological data and laboratory diagnosis. BN and AAL were the PIs for Asembo Bay Cohort Project and designed ABCP. LS and VU participated in design of the genetic association study and contributed to data interpretation. YPS was the PI for this genetic association study, designed the study and wrote manuscript. All authors read and approved the final manuscript.

## Supplementary Material

Additional file 1 Summary of p-value of associations between genotypes tested and malaria-associated morbidity. Click here for file
